# Chemical Characterization and In Vitro Evaluation of Glucans from Fermentation-Produced Nutraceutical Bionutri-AR1^®^: Antioxidant and Immunomodulatory Properties

**DOI:** 10.3390/pharmaceutics16111404

**Published:** 2024-10-31

**Authors:** Elaine R. Carbonero, Tammara S. M. Novikov, Yagly G. S. Gomes, Dayane R. Brito, Luisa C. Coelho, Marcia F. Mendes, Maria Carolina B. Di Medeiros Leal, Anamélia L. Bocca, Luciano M. Lião

**Affiliations:** 1 Instituto de Química, Universidade Federal de Catalão, Catalan 75704-020, GO, Brazil; 2 Departamento de Biologia Celular, Instituto de Ciências Biológicas, Universidade de Brasília, Brasilia 70910-900, DF, Brazilalbocca@unb.br (A.L.B.); 3 Departamento de Medicina, Centro Universitário do Planalto Central Apparecido dos Santos, Brasilia 72445-020, DF, Brazil; caroldimedeiros@gmail.com; 4 Plataforma Bi-Institucional de Pesquisa Translacional, Fundação Oswaldo Cruz, Ribeirao Preto 14040-900, SP, Brazil; 5 Instituto de Química, Universidade Federal de Goiás, Goiânia 74690-900, GO, Brazil; lucianoliao@ufg.br

**Keywords:** Bionutri-AR1^®^, glucans, structural characterization, antioxidant, immunomodulatory effects

## Abstract

**Background:** The consumption of nutraceuticals or food supplements has increased crucially, aiming to address nutrient deficits and enhance immune system function. To develop safe food products with unique nutritional and functional benefits, new production methods of these nutraceuticals such as the fermentative process have been gaining prominence for industrial applications. Bionutri-AR1^®^ is a nutraceutical produced via this bioprocess, featuring a complex composition, that has been used to improve the immune systems of debilitated people. **Objectives:** Considering the various biological properties attributed to glucans, one of its main components, this study aims to structurally characterize and evaluate, in vitro, the antioxidant and immunomodulatory potential of the polymers from this nutraceutical to assess whether these polymers contribute to the product’s reported biological effects. **Methods/Results:** Unlike previous reports, this study characterized by NMR, GC-MS, and Congo Red assay techniques two main glucans: a water-insoluble linear α-D-glucan with glycosidic bonds (1→4) and a soluble branched (1→3)- and (1→6)-linked β-glucan with a triple helix. Both glucans showed significant antioxidant activity, measured by their capacity to scavenge 2,2-diphenyl-1-picrylhydrazyl (DPPH) radicals. They were also capable of inducing the secretion of cytokines such as tumoral necrosis factor-alpha (TNF-α), interleukin 10 (IL-10), and interleukin 6 (IL-6), determined through capture enzyme-linked immunosorbent assay (ELISA), especially when co-stimulated with lipopolysaccharide (LPS). **Conclusions:** This suggests a dual action of these glucans in both proinflammatory and regulatory pathways. Future studies will describe the mechanisms by which these glucans, especially the insoluble ones, enhance immune system function, highlighting their potential use in immunotherapy.

## 1. Introduction

Nutraceuticals are dietary supplements designed to improve health by delivering a concentrated form of a food’s biologically active ingredient in a non-food matrix [1]. These products can be classified based on their chemical structure and nature, including carbohydrate derivatives, fatty acids, structural lipids, phenolic compounds, and isoprenoid derivatives [2,3,4].

These dietary supplements have been gaining prominence in the scientific community due to their natural origin, which ensures safety for consumption, affordability compared to conventional drugs, and a range of health benefits, such as antihypercholesterolemic [5,6], antioxidant [7,8], anticoagulant [9,10], antimicrobial [9,11], antiobesity [9,12], immunomodulatory [9,13], and anticachexia activities [14,15], among others.

Due to these factors, dietary interventions and the use of supplements have been gaining traction as effective methods for meeting nutritional needs and promoting health [4,16]. Consequently, the market and companies are increasingly exploring different procurement methods to develop new and innovative products [17,18].

Currently, fermentation is an important process for developing safe food products with unique nutritional and functional benefits [2,18]. This method has become an attractive bioprocess with potential application at industrial scale due to its advantages, including low energy requirements without compromising high production yields and minimal waste production [2,18]. One type of fermentation process involves using a solid matrix with no free water but sufficient moisture in the substrate to support the growth and metabolism of microorganisms. These microorganisms are capable of producing enzymes and, consequently, promoting the biosynthesis or accumulation of bioactive substances [2,18,19]. This methodology, known as solid-state fermentation, has been utilized to enhance the nutraceutical content of foods and agro-industrial by-products like barley, oats, soybean, wheat, rice, pineapple, and apple pomace, among others [2,18]. Dietary supplements can also be produced by a fermented grain food mixture, such as AOB^TM^, produced by AOA Japan Company (Higashiyotsugi, Tokyo, Japan.), which has demonstrated strong antioxidative effects and may serve as an adjuvant in the therapy of malignant neoplasia [20]. Another example is Bionutri AR1^®^, produced by Pharnutri R & D Indústria Alimentícia e Biotecnologia Ltd. (Rua Jacinto Scaglione, Conchal, Brazil) which features a complex composition of numerous nutrients, including vitamins, fatty acids (omegas 3 and 6), isoflavones, amino acids, and polysaccharides such as β-glucans [21,22].

According to reviews by Cruz et al. [21] and Fernandes Junior et al. [22], supplementation is essential for cancer patients undergoing chemo- and radiotherapy due to the side effects of these treatments, such as severe inflammatory processes, malnutrition, nausea, and emesis. They highlight that among the constituents of Bionutri AR1^@^, amino acids and β-glucans may be responsible for improving nutritional parameters and the overall condition of these patients, thereby mitigating the damage caused by the therapies.

The bioactivity of nutraceutical products is often associated with the carbohydrates present in their composition, especially glucans, which have several biological activities, including antioxidant, antimicrobial, anti-inflammatory, antiviral, anticancer, and immunomodulatory properties [23,24,25,26,27]. Due to their ability to act on the immune system by binding to specific receptors responsible for recognizing pathogens, like Toll-like receptors (TLRs) and Dectin-1, glucans are considered critical components of health and well-being, as this is the first barrier to disease [27,28,29]. A range of studies indicate that the recognition of particulate β-glucan-containing polysaccharides derived from *Saccharomyces cerevisiae* primarily occurs through the Dectin-1 receptor. This recognition mechanism is notably conserved across various species, including humans, mice, pigs, and bovines [27,30].

Glucans with immunomodulatory activity can be obtained from various sources, including bacteria, fungi, yeast, cereals, and plants. For example, curdlan, a β-glucan derived from bacteria, and OatβG, a cereal β-glucan derived from yeast or fungi, have shown similar capacities to interact with immune cell receptors and induce inflammatory activation [31]. These polymers comprise D-glucose (Glc) units linked through glycosidic bonds with α- or β-configurations. Their structures range from linear to branched, with differences in molar mass, solubility, and three-dimensional conformation [29]. Among bioactive glucans with immunomodulatory properties are those with α-configuration from the plant *Cyclocarya paliurus,* which have a main chain of α-D-Glc*p* (1→4) units branched at C-3 and C-6 [32]. A similar structure was found in the macrofungus *Morchella importuna*, with a backbone of α-1→4 and substitution only in C-6 [33]. In contrast, the yeast *Saccharomyces cerevisiae* produces a glucan with a main chain of β-Glc*p* (1→3) units and branches at C-6 [29]. Furthermore, from the sea urchin *Hemicentrotus pulcherrimus*, seven-branched glucans with a β-1,4 main chain were isolated, all replaced in C-6 [34]. These findings highlight the structural diversity of glucans and their potential relevance for immunomodulation applications. In addition to the monosaccharide composition, the variations in purification levels can elicit distinct biological responses. Research on the macrofungal *Inonotus obliquus* has demonstrated that the isolated polysaccharide fractions’ water solubility and acidic properties are critical determinants of their activity on pattern recognition receptors (PRRs), specifically TLR2 and TLR4 in macrophages. In contrast, such activity was not observed in the particulate fraction [35].

Considering the diverse biological properties attributed to glucans, this study aims to structurally characterize and evaluate, in vitro, the antioxidant and immunomodulatory potential of the polysaccharides from a fermentation-produced nutraceutical, Bionutri-AR1^@^, to determine whether these polymers contribute to the biological effects associated with this product.

## 2. Materials and Methods

### 2.1. Purification of the Glucans from Bionutri AR1^@^

**Figure 1:**
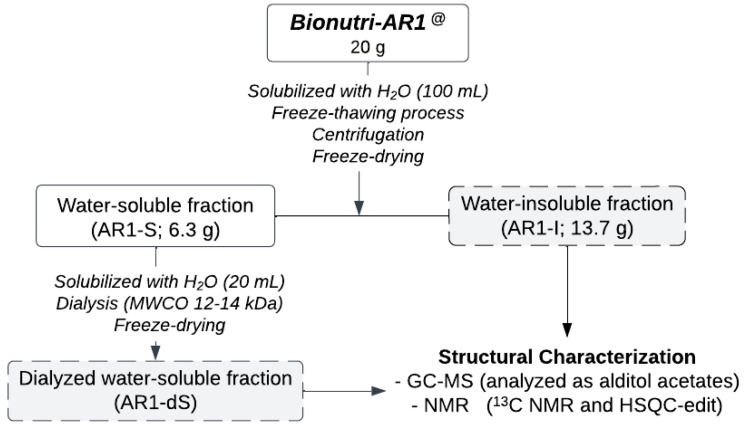
Scheme of purification and chemical analysis of polysaccharides from the Bionutri AR1^®^.

Firstly, distilled water (~100 mL) was added to an aliquot (20 g) of the Bionutri AR1^@^ fermented product, kindly provided by “Pharnutri R & D Indústria Alimentícia e Biotecnologia Ltd.”, and the mixture was heated and stirred magnetically for solubilization. After homogenization, the material was distributed in 50 mL Falcon tubes and submitted to the freeze/thaw fractionation process to separate the fractions according to solubility in cold water to ensure that all the insoluble material was separated from the soluble material. To do this, the material was frozen and then thawed at room temperature repeatedly until no insoluble material remained in the supernatant phase. Then, the cold-water-soluble portion (AR1-S) was separated from the insoluble portion (AR1-I) by centrifugation (5000 rpm, 10 min, 20 °C) and lyophilized (AR1-S: 6.3 g; AR1-I: 13.7 g). To separate the macromolecules from the soluble fraction (AR1-S), after solubilization in distilled water, the sample was dialyzed in a membrane with an exclusion limit of 12–14 kDa for approximately 24 h, concentrated under reduced pressure, and lyophilized (AR1-dS) (Figure 1).

### 2.2. Monosaccharide Composition

The monosaccharides in the fractions were identified following hydrolysis with 2 M trifluoroacetic acid (TFA) for 8 h at 100 °C, conversion to alditol acetates by successive NaBH_4_ reduction, and acetylation with Ac_2_O-pyridine (1:1, *v*/*v*) for 12 h at room temperature [36,37]. The alditol acetates obtained were analyzed by gas chromatography–mass spectrometry (GC-MS) using an Agilent model 3300 gas chromatograph (Agilent Technologies, Inc., Santa Clara, CA, USA) linked to a Finnigan Ion-Trap, Model 810-R12 mass spectrometer (Thermo Fisher Scientific Inc., Waltham, MA, USA). An HP5-MS capillary column (30 m × 0.25 mm i.d.) was used for qualitative and quantitative analysis of alditol acetates. The alditol acetates were identified by their typical retention times and electron impact profiles.

### 2.3. Nuclear Magnetic Resonance Analysis (NMR)

NMR spectra of carbon-13 (^13^C) NMR and edited heteronuclear single quantum correlation (HSQC-edit) were obtained using a Bruker Avance III 500 spectrometer (Bruker Corporation, Billerica, MA, USA) operated at 11.75 T (^1^H resonance frequency 500.13 MHz), equipped with a 5 mm broadband inverse (BBI) probe. Analyses were performed at 50 °C on samples dissolved in D_2_O (AR1-dS) or Me_2_SO-*d*_6_ (AR1-I). Chemical shifts are expressed in δ relative to Me_4_Si (TMS; δ = 0) or Me_2_SO-*d*_6_ (δ = 39.70 and 2.50 for ^13^C and ^1^H signals, respectively).

### 2.4. Triple-Helix Analysis

The conformational characteristics of the (1→3) (1→6) β-glucans isolated from Bionutri AR1^®^ (AR1-dS fraction) were evaluated by their interaction with Congo Red. Initially, a Congo Red solution (90 μmol/L) was equally mixed with an aqueous polysaccharide solution (2 mg/mL). The NaOH solution (3 M) was then added to the mixture to adjust the final NaOH concentration from 0.1 to 0.5 mol/L. At the same time, a Congo Red solution without polysaccharide was used as the experimental control group. Ultraviolet–visible (UV–vis) scanning was performed at 400 to 600 nm wavelengths, and the λ_max_ versus NaOH concentration diagram was then constructed [38].

### 2.5. Antioxidant Activity In Vitro

The antioxidant activity of the samples was measured using a 2,2-diphenyl-1-picrylhydrazyl (DPPH) assay [39]. The tested samples were prepared at different concentrations (0.2, 0.5, 1.0, 1.5, and 2.0 mg/mL). Briefly, DPPH methanol solution (100 uM) was mixed with polysaccharide samples dissolved in 5% DMSO aqueous solution at different concentrations and incubated at room temperature for 30 min in the dark. Then, the absorbance was measured at 517 nm using a UV spectrophotometer. The DPPH-scavenging activity was calculated using the following equation:

DPPH-scavenging rate (%) = [A_0_ − (A_2_ − A_1_)/A_0_] × 100; where A_0_ is the absorbance of the DPPH (without sample), A_2_ is that of the mixtures (sample + DPPH), and A_1_ is that without DPPH.

The half-maximal effective concentration (EC50) value of the antioxidant activity of the DPPH-scavenging method is defined as the effective antioxidant concentration required to reduce the initial DPPH concentration by 50%.

A calibration curve of ascorbic acid (5 to 50 μM) was made, and the EC50 value was determined.

### 2.6. Enzymatic and Acid Hydrolysis of the α-Glucan from Bionutri AR1^@^

The α-D-glucan (1→4) (AR1-I) (5 mg/mL) hydrolysis was carried out at 37 °C for 6 h under magnetic stirring. Experiments were conducted: (1) in the presence of α-amylase, and (2) in the presence of aq. HCl solution at pH 2.0. For both procedures, the polysaccharide solution (2 mg/mL) was added to the reaction medium and placed in an oven at 37 °C to carry out the hydrolysis.

### 2.7. Immunomodulatory Assay

The hematopoietic bone marrow cells from C57BL/6 mice were isolated from the tibia and femoral lavage with cold and sterile Roswell Park Memorial Institute (RPMI) 1640. After erythrocyte lysis, cells were differentiated into primary cultures of macrophages and dendritic cells (DCs) upon granulocyte–macrophage colony-stimulating factor (GM-CSF) stimulation as described previously [40,41]. The cell differentiation was assessed by flow cytometry, and an average of 79% of non-adherent cells were CD11c+/MHC class II+, whereas 81% of adherent cells were CD11b+/F4/80+, characterized as DCs and macrophages, respectively. Adherent (bone-marrow-derived macrophages—BMDMs) and non-adherent cells (bone marrow dendritic cells—BMDCs) were retrieved and transferred to 96-well plates at the final concentration of 1 × 10^6^/mL in RPMI 1640 (Gibco-Thermo Fisher Scientific) culture media with 10% fetal bovine serum (FBS). Both cell populations were either treated with *Escherichia coli* lipopolysaccharide (LPS) (500 ng/mL) or zymosan (100 mg/mL) as a positive control of cytokine production through TLR-4 and Dectin-1/TLR-2 receptors, respectively. The cells were also stimulated with AR1 samples (AR1, AR1-dS, and AR1-I) at concentrations of 25, 50, and 100 µg/ mL [42] or co-stimulated with LPS for incubated for 24 h at 37 °C, 5% CO_2_. The supernatant was collected for the determination of tumoral necrosis factor-alpha (TNF-α), interleukin 10 (IL-10), and interleukin 6 (IL-6) through capture enzyme-linked immunosorbent assay (ELISA), following the manufacturer’s instructions (Invitrogen^TM^, Waltham, MA, USA). High-affinity binding microwell plates (Corning^®^, Corning, NY, USA) were used. Results were expressed as pg/mL. All statistical analyses used one-way ANOVA.

## 3. Results and Discussion

### 3.1. Chemical Characterization of the Polysaccharides from Bionutri AR1^®^

Firstly, an aliquot (20 g) of the fermented product was solubilized in distilled water under magnetic stirring with heating and then subjected to fractionation by freezing/thawing, aiming at the fractionation of the molecules according to their solubility in cold water. This process yielded a cold-water-soluble fraction (AR1-S) and an insoluble fraction (AR1-I). After concentration of the volume under reduced pressure, if necessary, both fractions were lyophilized, resulting in a mass of 6.3 g for the soluble fraction and 13.7 g for the insoluble fraction, corresponding to approximately one-third of the soluble fraction to two-thirds of the insoluble fraction. To chemically analyze the macromolecules, it was necessary to perform dialysis of the soluble fraction (AR1-S) in membranes with an exclusion limit of 12–14 kDa, retaining only the carbohydrates of high molar mass, i.e., the polysaccharides, which were then lyophilized, resulting in the AR1-dS fraction (Figure 1).

To determine the monosaccharide composition of these fractions, an aliquot of each (2–5 mg) was hydrolyzed, reduced with sodium borohydride, and acetylated, resulting in the corresponding alditol acetates. These were then analyzed using gas chromatography–mass spectrometry (GC-MS).

**Figure 2:**
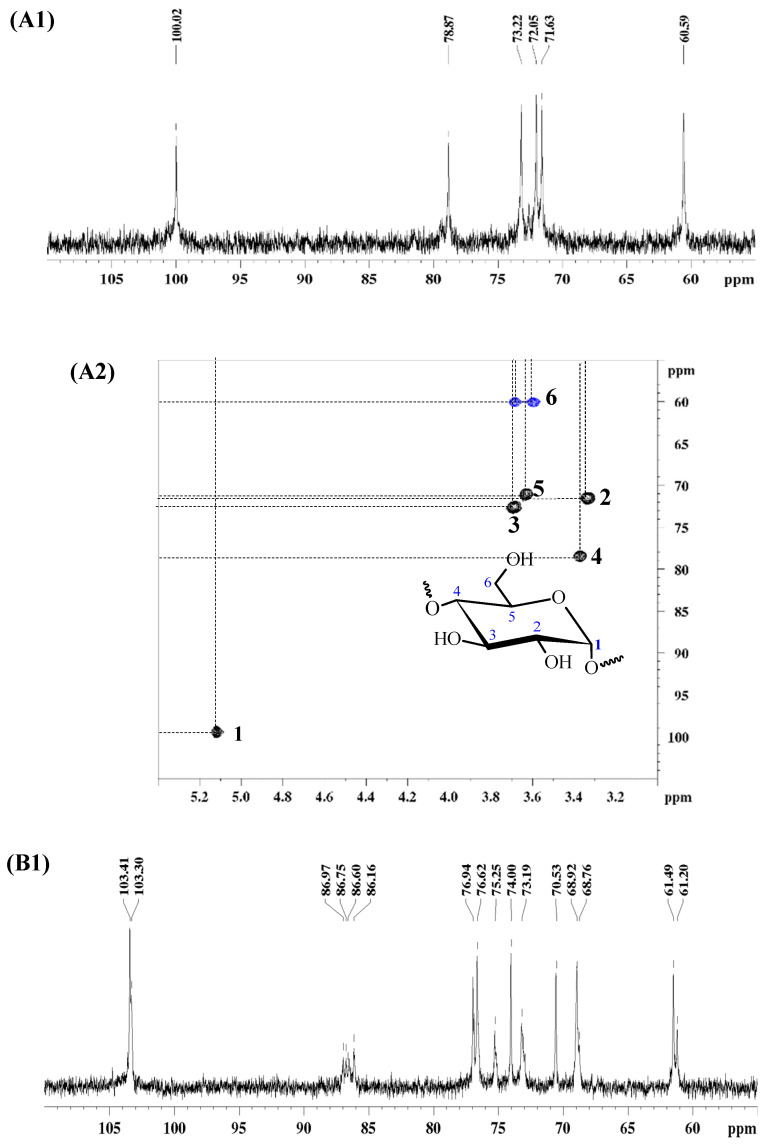

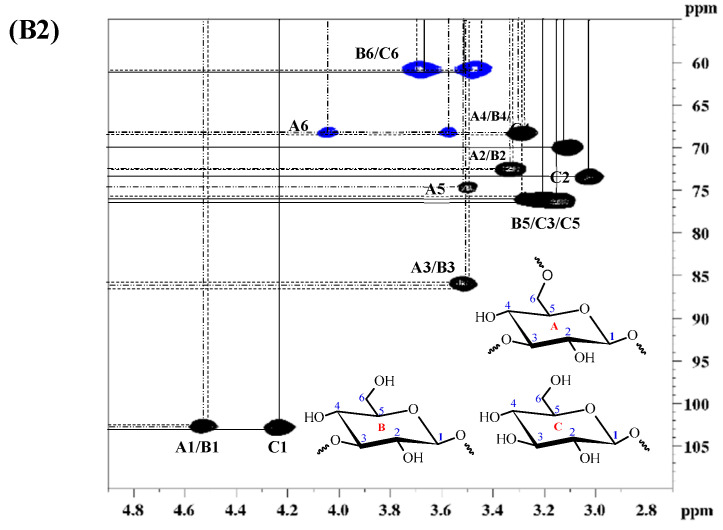
^13^C NMR and HSQC-edit spectra of AR1-I (**A1**,**A2**) and AR1-dS (**B1**,**B2**).

According to the GC-MS data, glucose (Glc) is the main monosaccharide, confirming the presence of glucans in these fractions. These fractions showed high molar masses (AR1-I: *M*_w_ 602 kDa; AR1-dS: *M*_w_ 1060 kDa), as determined by dynamic light scattering (DLS). For further information on the polymers present in these fractions, an aliquot (~30 mg) was subjected to carbon-13 nuclear magnetic resonance (^13^C NMR) and edited heteronuclear single quantum correlation (HSQC-edit) analysis (Figure 2).

**Figure 3:**
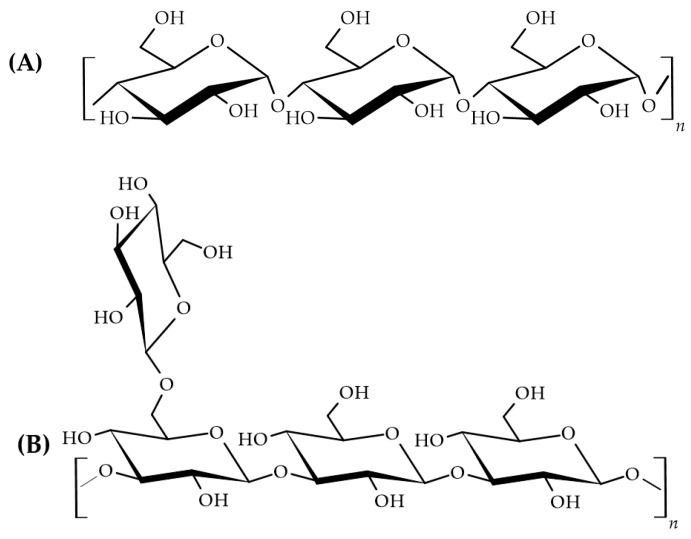
Proposed structures of glucans from Bionutri AR1^®^ ((**A**): α-D-Glucan 1→4; (**B**): β-D-Glucan 1→3, 1→6).

The ^13^C NMR spectra of the AR1-I (Figure 2A1) and AR1-dS fractions (Figure 2B1) were distinct, indicating the presence of different glucans. In the AR1-I fraction (Figure 2A1), the spectrum showed six main signals typical of a linear homopolymer formed by glucose, corresponding to an α-D-glucan with glycosidic linkages of type 1→4 (Figure 3A). The glycosidic configuration of type α was confirmed by the characteristics of C1/H1 signals at δ 100.02/5.12, and the glycosidic binding of type (1→4) by the presence of the substituted C-4/H-4 signals at δ 78.87/3.37 (Figure 2A2; Table 1) [43].

This α-D-glucan (1→4) was resistant to enzymatic hydrolysis with alpha-amylase and acid hydrolysis with aq. HCl at pH 2.0, both conducted at 37 °C. The resistance to degradation is probably due to its water insolubility, influenced by its high molecular weight and three-dimensional structure, among other factors.

In contrast, the NMR data of the AR1-dS fraction (Figure 2B1,B2; Table 1) were similar to those of the well-known branched β-(1→3)(1→6) glucan from macrofungi. The beta-glycosidic configuration was assigned due to the characteristics of C1/H1 signals at δ 102.82/4.52, 102.90/4.54, and 103.40/4.24. At the same time, the linkages of type (1→3) were suggested by the values between δ 86.16 and 86.97, corresponding to C-3 linkages, while those of type (1→6) were indicated by the presence of C-6 substituted at δ 68.49. This type of polymer consists of a repeating sequence formed by three types of units: β-D-Glc*p* units 3,6-di-*O*-(A), 3-*O*-substituted (B), and non-reducing ends of β-D-Glc*p* units (C). All ^13^C and ^1^H assignments of the glucan in the AR1-dS fraction were determined by the HSQC-edit spectrum (Figure 2B2; Table 1) and are consistent with literature data for similar polymers [44]. According to the data, the AR1-dS fraction corresponds to a glucan with a β-D-Glc*p* (1→3) main chain, partially substituted at O-6 by non-reducing terminals of β-D-Glc*p*, according to the structure proposed in Figure 3B.

**Figure 4:**
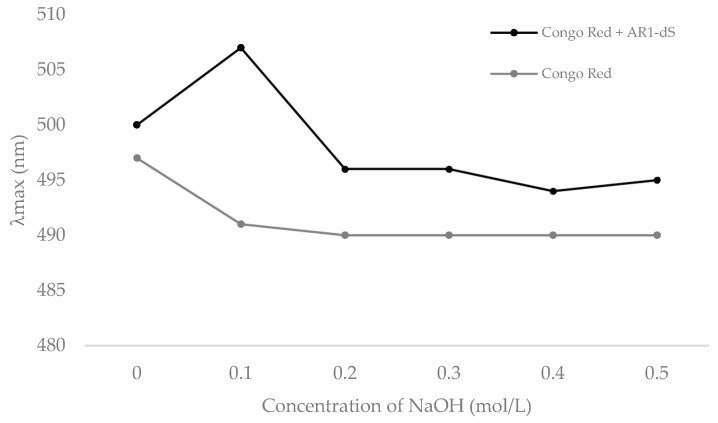
Changes in maximum absorption of Congo Red + AR1-dS complex at various concentrations of NaOH.

Due to their capacity, beta-glucans with a triple-helix structure could form characteristic complexes with Congo Red; the AR1-dS was treated with this dye in the presence of different concentrations of NaOH to verify the tridimensional conformation. The interactions between AR1-dS and Congo Red (Figure 4) increased the absorption maxima (λ_max_) value in aqueous or weakly alkaline solutions. In contrast, a decrease in the λ_max_ was observed in highly alkaline concentrations. It can be concluded that AR1-dS possesses a relatively ordered triple helical conformation, which was destroyed with a further rise in NaOH solution concentration.

The purified beta-glucan is similar to those described for macrofungi, such as those isolated from *Lentinula edodes* (known as “*Lentinan*”), *Schizophyllum commune* (“*Schizophyllan*”), *Auricularia auricula-judae* (“*Auricularian*”), which have been gaining scientific importance due to the proof of their biological properties, one of which is their ability to act as an immunomodulator. This function has been closely associated with their triple-helix conformation, improving the defense response, especially in debilitated patients [38].

Surprisingly, the amylose-like alpha-1,4-glucan, which is in vitro enzymatic and acid hydrolysis resistant, is the major polysaccharide in Bionutri AR1^®^ and may have health benefits.

Previous studies have found that resistant starch, mainly types with high amylose, can affect food intake, satiety, body weight and composition, glucose and insulin response, blood lipid profiles, inflammation, oxidative status, and intestinal microorganisms and the health of the intestines [45,46,47]. This is primarily achieved through the production of essential metabolites, especially short-chain fatty acids (SCFAs), which play a crucial role in improving physical and mental health [46].

### 3.2. Antioxidant Activity of the Glucans from Bionutri AR1^®^

The antioxidant capacity of the commercial product Bionutri AR1^@^ (AR1) and its fractions, AR1-I and AR1-dS, was evaluated by scavenging 2,2-diphenyl-1-picrylhydrazyl (DPPH) free radicals. This was achieved by measuring the decrease in its absorbance at 517 nm, which occurred when a hydrogen atom or an electron was captured by DPPH radical scavenging, resulting in a color change of DPPH from purple to pale yellow.

**Figure 5:**
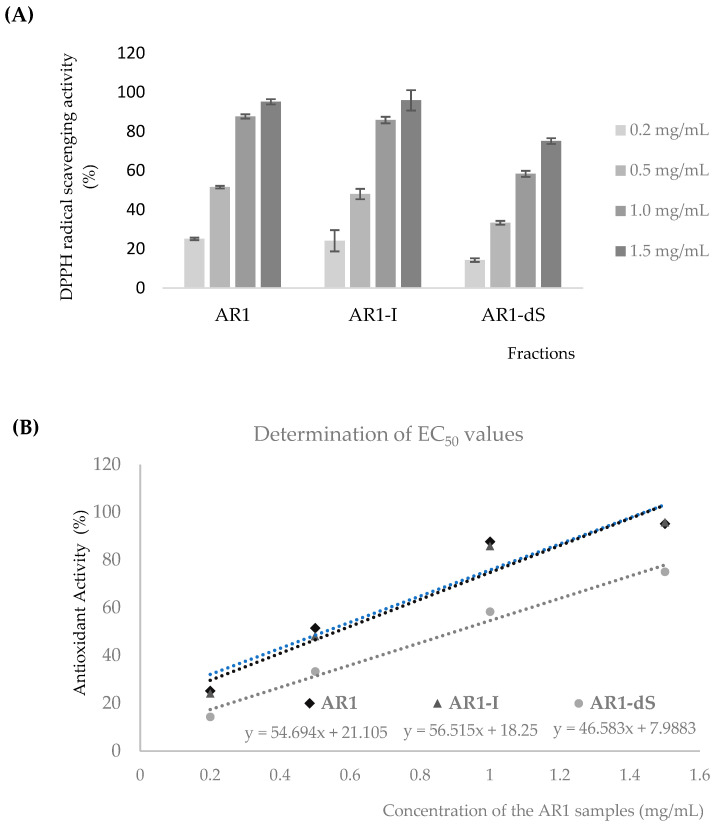
DPPH-radical-scavenging rate (**A**) and EC_50_ value determination (**B**) of AR1 samples (AR1, AR1-dS, and AR1-I) at different concentrations. The data are the means of 3 independent measurements ± standard deviations.

Our results demonstrate a concentration-dependent scavenging activity of AR1, AR1-I, and AR1-dS on DPPH free radicals within the 0.2 to 1.0 mg/mL range (Figure 5A). The half-maximal effective concentration (EC50) values (Figure 5B), indicating the concentration of an antioxidant-containing substance required to scavenge 50% of the initial DPPH radicals, were 0.53, 0.56, and 0.90 mg/mL for AR1, AR1-I, and AR1-dS, respectively. Notably, the antioxidant activity of the α-glucan fractions (AR1 and AR1-I) was found to be significantly superior to that of β-glucan, underscoring the potential of these (1→4)-linked α-glucans from the Bionutri-AR1^®^ nutraceutical.

### 3.3. Immunomodulatory Effects of the Glucans from Bionutri AR1^®^

**Figure 6:**
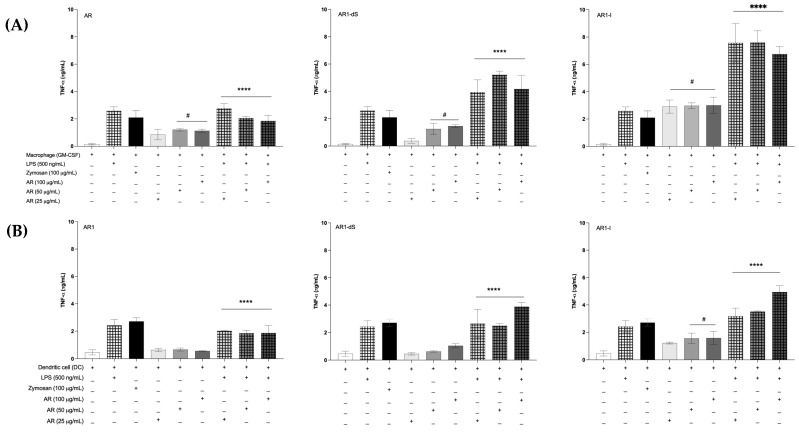
TNF-α secretion by bone-marrow-derived macrophages (BMDMs) (**A**) and bone marrow dendritic cells (BMDCs) (**B**) after stimulation with AR1 samples (AR1, AR1-dS, and AR1-I) at different concentrations or co-stimulated with lipopolysaccharide (LPS). As controls, cells were stimulated with LPS and Zymosan. The data are the means of three independent measurements ± standard deviations. # *p* < 0.05 compared to non-stimulated cells; **** *p* < 0.01 compared with cells stimulated with glucans. All statistical analyses used one-way ANOVA.

To evaluate the capacity of the glucans to stimulate an inflammatory response, bone-marrow-derived macrophages (BMDMs) and bone marrow dendritic cells (BMDCs) were differentiated from hematopoietic cells, as previously described by Silva et al. [40]. The AR1-I sample increased the tumor necrosis factor-alpha (TNF-α) production by BMDMs in all concentrations tested, while AR1 and AR1-dS achieved this only at higher concentrations, compared with the non-stimulated cells. Stimulation with lipopolysaccharide (LPS) plus the glucans induced higher concentrations of this cytokine when compared with the cells stimulated only with LPS, especially when stimulated by AR1-I and AR1-dS (Figure 6A). However, the AR1 and AR1-dS samples did not stimulate the BMDCs to produce TNF-α; only AR1-I (Figure 6B) showed this effect. As observed in BMDMs, the stimulation with LPS increased the production of this cytokine when compared with cells stimulated with glucans; however, there is no increased production of this cytokine for the groups stimulated with AR1 and the lower concentrations of AR1-dS when compared with cells stimulated only with LPS (Figure 6B). Considering the low cytokine production by BMDCs, we did not measure the other cytokines secreted by dendritic cells.

**Figure 7:**
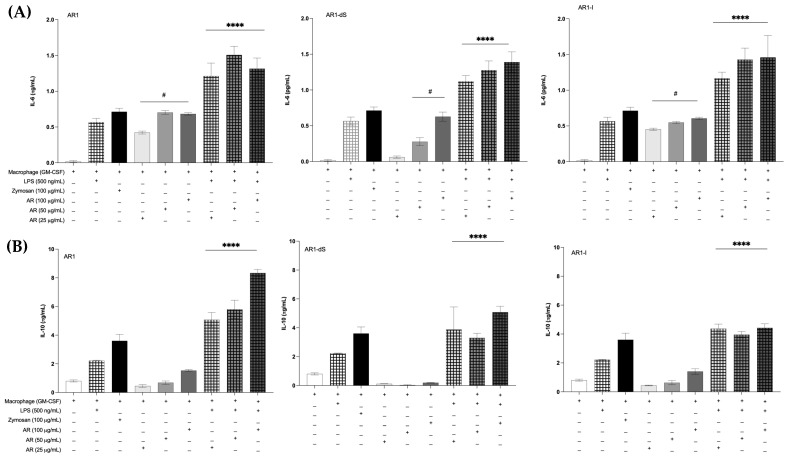
IL-6 (**A**) and IL-10 (**B**) secretion by bone-marrow-derived macrophages (BMDMs) after stimulation with AR1 samples (AR1, AR1-dS, and AR1-I) at different concentrations or co-stimulated with LPS. As a control, the cells were stimulated with LPS and Zymosan. The data are the means of three independent measurements ± standard deviations. # *p* < 0.05 compared with non-stimulated cells; **** *p* < 0.01 compared with cells stimulated with glucans. All statistical analysis used one-way ANOVA.

Another essential inflammatory cytokine is interleukin-6 (IL-6). The capacity of the samples to stimulate the secretion of IL-6 was the same when the BMDMs were stimulated with AR1 and AR1-I. The co-stimulation with LPS showed similar levels of IL-6 when stimulated with all samples (Figure 7A). As expected, the secretion of interleukin-10 (IL-10) was not inducted by the glucan samples only if the LPS stimuli were added to the cells (Figure 7B). Notably, AR1 showed higher levels of IL-10 after stimulation with LPS.

These data indicate that immune cells retain their ability to respond to stimuli beyond glucan exposure. Furthermore, glucan does not inhibit cellular responses mediated by other receptors or intracellular pathways nor induce negative stimuli that compromise cellular function.

The importance of proinflammatory cytokines in the innate immune response is extensively documented [48]. Nevertheless, the ultimate effects of these cytokines are contingent upon the surrounding inflammatory environment. For instance, in colorectal cancer, interleukin-6 (IL-6) and tumor necrosis factor-alpha (TNF-α) are implicated in promoting cancer cell invasion and fostering the formation of tumor-associated stroma through mechanisms regulated by signal transducer and activator of transcription 3 (STAT3) [49]. Likewise, IL-10 plays a double role during the immune response activation. This cytokine is critical in modulating inflammation and maintaining cell homeostasis, especially in hyperinflammatory pathologies, including cancer or infectious diseases [50]. Notably, IL-10 possesses immunostimulating properties because it potentiates B lymphocytes, enhancing B cell growth, proliferation, and activation and driving differentiation into immunoglobulin-secreting plasma cells. Additionally, IL-10 promotes the survival of T cells otherwise destined for apoptotic cell death, stimulates NK cell proliferation and migration, augments their cytolytic activity and effector functions, and acts as a cytotoxic differentiation factor. Furthermore, it promotes IL-2-driven proliferation and differentiation of precursor CD8+ splenocytes into effector CTL [50]. To elucidate the interactions between these cytokines under various inflammatory conditions, further in vivo investigations are warranted.

The immunomodulatory activity of glucans has been described by different sources and compositions [27].

Here, all AR1 samples can induce different levels of inflammatory cytokines, and the insoluble ones (α-glucans) showed higher secretion of inflammatory cytokines. Glucans, with α- or β-configuration, can activate innate immune response through cell maturation and cytokine secretion and increase phagocytosis. Additionally, they can regulate the adaptive immune response as the antigen-presenting cells process and immune tolerance. The inflammatory potential to induce cytokine production is better described for insoluble β-glucan, which can interact with Dectin-1 [51]. When the structure of β-glucan is associated with other molecules such as lipids, mannoproteins, and chitins, the molecule can interact with complement receptor 3 (CR3) and Toll-like receptor 2 (TLR-2), stimulating different intracellular pathways [27,52]. The α-glucan from *Lomentospora prolificans* can modulate the host response through Dectin-1 and Mincle [53] but not through TLR-2 and TLR-4 [54]. The ability of α-glucans to bind to PRRs with different affinities depends on their structure [55,56].

The glucans, especially those with a β-configuration in their composition, interact with Dectin-1 and increase cytokine secretion throughout the Syk/Card9/NF-kB pathway [31]. Purified β-glucan has been observed to activate the Dectin-1/Raf-1/mTOR signaling axis [52], showing the effects of receptor crosstalk when cells are stimulated with different glucan compositions.

All these activities occur without excessive inflammation, triggering tissue damage, depending on glucan types, that can be significantly influenced by their sources, such as water solubility, molecular weight, degree of branching, and tridimensional structure [57,58], which directly influence the affinity of these polymers for the receptor [27,55,56].

Although numerous reports confirm that the chemical structure of glucans influences the immune response, there is still no consensus regarding the essential characteristics that make them more effective due to the different specificities of the receptors that recognize them [57]. Regarding β-glucans, it is known that the higher-molecular-weight glucans and triple-helix conformation have more effect on the immune system. It has also been reported that the β-(1,3)-D-glucopyranosyl backbone of the glucans, with at least seven glucose units, is essential for Dectin-1 recognition [57,58]. The side chain length and branching frequency are also crucial for the immunomodulating ability of β-glucans, being more active in those with branching degrees ranging from 0.20 (1:5 branching) to 0.33 (1:3 branching) [57,58]. It is important to emphasize that the methodology of extraction and purification of these polymers, as well as the drying process (freeze-drying, spray dryer, vacuum oven), can cause changes in their physical properties (solubility, viscosity, gelling capacity, three-dimensional structure, among others) and consequently influence their biological functions, making it difficult to correlate the structure versus biological activity.

The interaction of macrophages and all types of glucans tested spontaneously induced TNF-α and IL-6 in an interesting way. The levels of these cytokines after co-stimulation with LPS increased highly, suggesting a distinct PRR interaction of glucans and LPS, which activated the cytokine production through TLR-4. This indicates that glucans can enhance the immune response, potentially aiding in combating infections where inflammation is critical for pathogen clearance, in both bacterial [59] and fungal infections [60]. The interaction of dendritic cells with the glucans only increased the proinflammatory cytokines in the higher concentrations. Still, in all concentrations, LPS acts additively to the glucans, probably through the same PRR of macrophages. This differential response between macrophages and dendritic cells highlights the specificity of glucan interactions with different immune cell types. Interestingly, IL-10 secretion, which typically indicates anti-inflammatory responses, was induced by all AR1 samples after LPS stimulation. This indicates that these glucans preserved the cell potential to activate both proinflammatory and regulatory pathways, and it will be dependent on the other stimuli in the milieu.

Glucans, mainly β-glucan, are classified as biological response modifiers (BRMs), improving human health. This group of molecules acts as immunomodulators, targeting disease-causing mechanisms. Several studies have shown that glucans enhance dendritic cell maturation and cytokine secretion and regulate adaptive immune response. These activities are associated with glucan receptors, especially Dectin-1, which operate in various tissues [27,61]. Given these properties, glucans were extensively tested and evaluated in cancer patients, reducing cancer development and alleviating immune suppression in the cancer milieu. Additionally, they have shown evidence of being anticytotoxic and antimutagenic [62]. Due to their anticancer properties, the molecules analyzed here are considered potential candidates for use as pharmaceutical health promoters. The underlying action mechanisms involve the release of cytokines, which activate antitumor immune cells as macrophages, enhancing proinflammatory cytokine production and phagocytic activity, thereby strengthening immune responses. Our results indicate that AR1-I can especially induce TNF-α and IL-6 production by macrophages and dendritic cells. So, we speculate that linear (1→4)-linked α-glucan may have significant potential as an adjuvant in cancer treatment, mainly for patients with malnutrition caused by the collateral effects of chemo- and radiotherapy, and can have an important role to improve the therapeutic effects and the quality of life these patients.

Our data corroborate other publications, demonstrating that the insoluble sample is more efficient at inducing proinflammatory cytokines at lower concentrations and achieving higher levels when co-stimulated with LPS. As discussed earlier, the mechanism by which beta-glucans act on the immune system is well known. Although there are reports of α-glucans with immunomodulatory properties, most of them do not mention the pathway and receptors of the immune cells responsible for these effects. In this way, the mechanisms by which these glucans enhance the immune system will be described in ongoing studies, but they have the potential to be used in the future as immunotherapy.

## 4. Conclusions

Bionutri AR1^®^ contains high levels of glucans, especially the water-insoluble linear alpha-glucan with (1→4) linkages, which is resistant to acid and enzymatic hydrolysis (amylase). This finding contrasts with previous colorimetric methods that identified only β-glucan. Surprisingly, both were efficient in the scavenging of 2,2-diphenyl-1-picrylhydrazyl (DPPH) free radicals, evidencing the antioxidant action. They were also capable of inducing the secretion of the cytokines TNF-α, IL-6, and IL-10, especially when co-stimulated with LPS. This suggests a dual action of these glucans in both proinflammatory and regulatory pathways, potentially improving immune system health in debilitated people, such as patients undergoing chemo- or radiotherapy, who experience severe side effects on inflammatory processes. Due to the findings in this research regarding the ability of α-glucans to activate the immune response, future studies are needed to determine the receptors involved in their recognition, as well as the mechanisms of action, since there are few studies involving glucans with this type of glycosidic configuration.

## Figures and Tables

**Table 1:** Assignments of ^13^C and ^1^H of the glucans from Bionutri AR1^@^ *.

Fractions	Units		1	2	3	4	5	6	
								6a	6b
AR1-I	→4)-α-Glc*p-*(1→	^13^C	***100.02***	72.05	73.22	***78.87***	71.63	60.59	
		^1^H	***5.12***	3.33	3.69	***3.37***	3.63	3.68	3.60
AR1-dS	→3,6)-β-Glc*p-*(1→ **(A)**	^13^C	***102.90***	72.79	***86.16***	68.47	74.85	***68.49***	
		^1^H	***4.54***	3.34	***3.50***	3.28	3.50	***4.04***	3.47
	→3)-β-Glc*p-*(1→ **(B)**	^13^C	***102.82***	72.47	***86.97/86.75/86.60***	68.68	76.25	60.84	
		^1^H	***4.52***	3.32	***3.51***	3.26	3.28	3.69	3.47
	β-Glc*p-*(1→ **(C)**	^13^C	***103.40***	73.70	76.43	70.27	76.56	61.16	
		^1^H	***4.24***	3.02	3.20	3.10	3.15	3.68	3.49

* Based ^13^C NMR and HSQC-edit spectra. The assignments of carbon and hydrogen highlighted in italics and bold refer to those involved in glycosidic linkages.

## Data Availability

The original contributions presented in this study are included in the article. Further inquiries can be directed to the corresponding author.

## References

[1] Zeisel S.H. (1999). Regulation of “Nutraceuticals”. Science.

[2] Postigo L.O.C., Jacobo-Velázquez D.A., Guajardo-Flores D., García-Cayuela T. (2021). Solid-state fermentation for enhancing the nutraceutical content of agrifood by-products: Recent advances and its industrial feasibility. Food Biosci..

[3] Santana-Gálvez J., Cisneros-Zevallos L., Jacobo-Velázquez D.A. (2019). A practical guide for designing effective nutraceutical combinations in the form of foods, beverages, and dietary supplements against chronic degenerative diseases. Trends Food Sci. Technol..

[4] Sachdeva V., Roy A., Bharadvaja N. (2020). Current prospects of nutraceuticals: A review. Curr. Pharm. Biotechnol..

[5] Sut S., Dall’Acqua S. (2023). Food-derived nutraceuticals for hypercholesterolemia management, mode of action and active ingredients. Food Biosci..

[6] Santini A., Novellino E. (2017). Nutraceuticals in hypercholesterolaemia: An overview. Br. J. Pharmacol..

[7] Kamran F., Phillips M., Harman D., Reddy N. (2023). Antioxidant activities of lupin (*Lupinus angustifolius*) protein hydrolysates and their potential for nutraceutical and functional foods. Food Chem. Adv..

[8] Ramli N.Z., Yahaya M.F., Tooyama I., Damanhuri H.A. (2020). A mechanistic evaluation of antioxidant nutraceuticals on their potential against age-associated neurodegenerative diseases. Antioxidants.

[9] Devi P.V., Islam M.J., Narzary P., Sharma D., Siltana F. (2024). Bioactive compounds, nutraceutical values and its application in food product development of oyster mushroom. J. Future Foods.

[10] Minno A.D., Frigerio B., Spadarella G., Ravani A., Sansaro D., Amato M., Kitzmiller J.P., Pepi M., Tremoli E., Baldassarre D. (2017). Old and new oral anticoagulants: Food, herbal medicines and drug interactions. Blood Rev..

[11] Gutiérrez-Del-Río I., Fernández J., Lombó F. (2018). Plant nutraceuticals as antimicrobial agents in food preservation: Terpenoids, polyphenols and thiols. Int. J. Antimicrob. Agents.

[12] Abdelfattah D.S.E., Fouad M.A., Elmeshad A.N., Nabarawi M.A., Elhabal S.F. (2024). Anti-obesity nutraceuticals: Insights into mechanisms of action and potential use of biocompatible nanocarriers for delivery. Int. J. Appl. Pharm..

[13] Medoro A., Davinelli S., Colletti A., Micoli V.D., Grandi E., Fogacci F., Scapagnini G., Cicero A.F.G. (2023). Nutraceuticals as modulators of immune function: A review of potential therapeutic effects. Prev. Nutr. Food Sci..

[14] Caeiro L., Gandhay D., Anderson L.J., Garcia J.M. (2023). A review of nutraceuticals in cancer cachexia. Cancers.

[15] Aquila G., David A., Cecconi R., Brault J.J., Corli O., Piccirillo R. (2020). Nutraceuticals and exercise against muscle wasting during cancer cachexia. Cells.

[16] Chopra A.S., Lordan R., Horbanczuk O.K., Atanasov A.G., Chopra I., Horbanczuk J.O., Józwik A., Huang L., Pirgozliev V., Banach M. (2022). The current use and evolving landscape of nutraceuticals. Pharmacol. Res..

[17] Otles S., Gokgunnec L., Smithers G.W. (2023). Safety considerations in developing functional foods and nutraceuticals. Encyclopedia of Food Safety.

[18] Tsafrakidou P., Michaelidou A.M., Biliaderis C.G. (2020). Fermented cereal-based products: Nutritional aspects, possible impact on gut microbiota and health implications. Foods.

[19] Behera S.S., Ray R.C. (2016). Solid state fermentation for production of microbial cellulases: Recent advances and improvement strategies. Int. J. Biol. Macromol..

[20] Minamiyama Y., Takemura S., Hirohashi K., Okada S. (2004). A fermented grain food mixture, AOB^TM^, inhibits liver metastasis in the metastasis model of rat colon cancer. Biofactors.

[21] Cruz E.M.S., Fernandes Junior H.J., Tallo F.S., Pires-Oliveira M., Nicolau A.L.D., Carvalho R.G., Gehrke F.S., Caricati-Neto A., Taha M.O., Rodrigues F.S.M. (2022). Benefits promoted by the use of a highly bioavailable fermentation-produced nutraceutical, rich in β-glucans and amino acids, for cancer patients treated with chemotherapy and radiotherapy. Res. Soc. Dev..

[22] Fernandes Junior H.J., Tallo F.S., Góes R.B., Oliveira C.T.F., Nicolau L.A.D., Arias A.N., Viana B.L.A., Menezes-Rodrigues F.S. (2024). Potential clinical benefits and probable mechanisms of action promoted by a nutraceutical obtained by fermentation and rich in β-glucans and amino acids for oncologic patients. J. Med. Resid. Rev..

[23] Alves Junior R.S., Silva C.A., Santos C., Nascimento V.M.G., Toledo K.A. (2024). The impacts of the immunomodulatory effects of fungal beta-glucans on human health. Braz. J. Health Rev..

[24] Cerletti C., Esposito S., Iacoviello L. (2021). Edible mushrooms and beta-glucans: Impact on human health. Nutrients.

[25] Ciecierska A., Drywień M.E., Hamulka J., Sadkowski T. (2019). Nutraceutical functions of beta-glucans in human nutrition. Rocz. Panstw. Zakl. Hig..

[26] Sengül M., Ufuk S. (2022). Therapeutic and functional properties of beta-glucan and its effects on health. Eurasian J. Food Sci. Technol..

[27] Zhong X., Wang G., Li F., Fang S., Zhou S., Ishiwata A., Tonevitsky A.G., Shkurnikov M., Cai H., Ding F. (2023). Immunomodulatory effect and biological significance of β-glucans. Pharmaceutics.

[28] Enshasy H.A.E., Kaul R.H. (2013). Mushroom immunomodulators: Unique molecules with unlimited applications. Trends Biotechnol..

[29] Jesus S., Costa J.P., Colaço M., Lebre F., Mateus D., Sebastião A.I., Cruz M.T., Alfaro-Moreno E., Borges O. (2024). Exploring the immunomodulatory properties of glucan particles in human primary cells. Int. J. Pharm..

[30] Pedro A.R.V., Lima T., Fróis-Martins R., Leal B., Ramos I.C., Martins E.G., Cabrita A.R.J., Fonseca A.J.M., Maia M.R.G., Vilanova M. (2021). Dectin-1-mediated production of pro-inflammatory cytokines induced by yeast β-glucans in bovine monocytes. Front. Immunol..

[31] Graaff P., Berrevoets C., Rösch C., Schols H.A., Verhoef K., Wichers H.J., Debets R., Govers C. (2021). Curdlan, zymosan and a yeast-derived β-glucan reshape tumor-associated macrophages into producers of inflammatory chemo-attractants. Cancer Immunol. Immunother..

[32] He S., Yan J., Chen L., Chen H., Wan W. (2024). Structure and in vitro antioxidant and immunomodulatory activity of a glucan from the leaves of *Cyclocarya paliurus*. J. Funct. Foods.

[33] Wen Y., Bi S., Hu X., Yang J., Li C., Li H., Yu D.B., Zhu J., Song L., Yu R. (2021). Structural characterization and immunomodulatory mechanisms of two novel glucans from *Morchella importuna* fruiting bodies. Int. J. Biol. Macromol..

[34] Shang A., Jiang Y., Yang F., Wu F., Zheng G., Lin Y., Wang C., Xin W., Zhao F. (2024). A homologous series of α-glucans from *Hemicentrotus pulcherrimus* and their immunomodulatory activity. Int. J. Biol. Macromol..

[35] Wold C.W., Christopoulos P.F., Arias M.A., Dzovor D.E., Øynebråten I., Corthay A., Inngjerdingen K.T. (2024). Fungal polysaccharides from *Inonotus obliquus* are agonists for Toll-like receptors and induce macrophage anti-cancer activity. Commun. Biol..

[36] Wolfrom M.L., Thompson A. (1963). A reduction with sodium borohydride. Methods Carbohydr. Chem..

[37] Wolfrom M.L., Thompson A. (1963). Acetylation. Methods Carbohydr. Chem..

[38] Guo X., Kang J., Xu Z., Guo Q., Zhang L., Ning H., Cui S.W. (2021). Triple-helix polysaccharides: Formation mechanisms and analytical methods. Carbohydr. Polym..

[39] Molyneux P. (2004). The use of the stable free radical diphenylpicrylhydrazyl (DPPH) for estimating antioxidant activity. Songklanakarin J. Sci. Technol..

[40] Silva G.S., Silva D.A., Guilhelmelli F., Jerônimo M.S., Cardoso-Miguel M.R.D., Bürgel P.H., Castro R.J.A., de Oliveira S.A.M., Silva-Pereira I., Bocca A.L. (2021). Zymosan enhances in vitro phagocyte function and the immune response of mice infected with *Paracoccidioides brasiliensis*. Med. Mycol..

[41] Lutz M.B., Kukutsch N., Ogilvie A.L.J., Rössner S., Koch F., Romani N., Schuler G. (1999). An advanced culture method for generating large quantities of highly pure dendritic cells from mouse bone marrow. J. Immunol. Methods.

[42] Basso A.M.M., De Castro R.J.A., De Castro T.B., Guimarães H.I., Polez V.L.P., Carbonero E.R., Pomin V.H., Hoffmann C., Grossi-de-Sa M.F., Tavares A.H. (2020). Immunomodulatory activity of β-glucan-containing exopolysaccharides from *Auricularia auricular* in phagocytes and mice infected with *Cryptococcus neoformans*. Med. Mycol..

[43] Falk H., Stanek M. (1997). Two-dimensional ^1^H and ^13^C NMR spectroscopy and the structural aspects of amylose and amylopectin. Monatshefte Chem..

[44] Kono H., Kondo N., Hirabayashi K., Ogata M., Totani K., Ikematsu S., Osada M. (2017). NMR spectroscopic structural characterization of a water-soluble β-(1→3,1→6)-glucan from *Aureobasidium pullulans*. Carbohydr. Polym..

[45] Guo J., Tan L., Kong L. (2022). Multiple levels of health benefits from resistant starch. J. Agric. Food Res..

[46] Li C., Dhital S., Gidley M.J. (2023). High amylose wheat foods: A new opportunity to improve human health. Trends Food Sci. Technol..

[47] Li H.-T., Zhang W., Zhu H., Chao C., Guo Q. (2023). Unlocking the potential of high-amylose starch for gut health: Not all function the same. Fermentation.

[48] Albrecht L.J., Tauber S.C., Merres J., Kress E., Stope M.B., Jansen S., Pufe T., Brandenburg L.O. (2016). Lack of proinflammatory cytokine interleukin-6 or tumor necrosis factor receptor-1 results in a failure of the innate immune response after bacterial meningitis. Mediators Inflamm..

[49] Chung S.S., Wu Y., Okobi Q., Adekoya D., Atefi M., Clarke O., Dutta P., Vadgama J.V. (2017). Proinflammatory cytokines IL-6 and TNF-α increased telomerase activity through NF-κB/STAT1/STAT3 activation, and Withaferin A inhibited the signaling in colorectal cancer cells. Mediators Inflamm..

[50] Carlini V., Noonan D.M., Abdalalem E., Goletti D., Sansone C., Calabrone L., Albini A. (2023). The multifaceted nature of IL-10: Regulation, role in immunological homeostasis and its relevance to cancer, COVID-19 and post-COVID conditions. Front Immunol..

[51] Marakalala M.J., Williams D.L., Hoving J.C., Engstad R., Netea M.G., Brown G.D. (2013). Dectin-1 plays a redundant role in the immunomodulatory activities of β-glucan-rich ligands in vivo. Microbes Infect..

[52] Cheng Q.J., Farrell K., Fenn J., Ma A., Makanani S.K., Siemsen J. (2024). Dectin-1 ligands produce distinct training phenotypes in human monocytes through differential activation of signaling networks. Sci. Rep..

[53] Xisto M.I.D.S., Dias L.S., Bezerra F.F., Bittencourt V.C.B., Rollin-Pinheiro R., Cartágenes-Pinto A.C., Haido R.M.T., Mourão P.A.D.S., Barreto-Bergter E. (2023). An alpha-glucan from *Lomentospora prolificans* mediates fungal–host interaction signaling through Dectin-1 and Mincle. J. Fungi.

[54] Felice B., Damiano S., Montanino C., Buono A., Rosa G., Guida B., Santillo M. (2020). Effect of beta- and alpha-glucans on immune modulating factors expression in enterocyte-like Caco-2 and goblet-like LS 174T cells. Int. J. Biol. Macromol..

[55] Noss I., Doekes G., Thorne P.S., Heederik D.J.J., Wouters I.M. (2013). Comparison of the potency of a variety of β-glucans to induce cytokine production in human whole blood. Innate Immun..

[56] Adams E.L., Rice P.J., Graves B., Ensley H.E., Yu H., Brown G.D., Gordon S., Monteiro M.A., Papp-Szabo E., Lowman D.W. (2008). Differential high-affinity interaction of dectin-1 with natural or synthetic glucans is dependent upon primary structure and is influenced by polymer chain length and side-chain branching. J. Pharmacol. Exp. Ther..

[57] Han B., Baruah K., Cox E., Vanrompay D., Bossier P. (2020). Structure-functional activity relationship of β-glucans from the perspective of immunomodulation: A mini-review. Front. Immunol..

[58] Bohn J.A., BeMiller J.N. (1995). (1→3) β-D-Glucans as biological response modifiers: A review of structure-functional activity relationships. Carbohydr. Polym..

[59] Geng J., Shi Y., Zhang J., Yang B., Wang P., Yuan W., Zhao H., Li J., Qin F., Hong L. (2021). TLR4 signalling via Piezo1 engages and enhances the macrophage mediated host response during bacterial infection. Nat. Commun..

[60] Onyishi C.U., Desanti G.E., Wilkinson A.L., Lara-Reyna S., Frickel E.M., Fejer G., Christophe O.D., Bryant C.E., Mukhopadhyay S., Gordon S. (2023). Toll-like receptor 4 and macrophage scavenger receptor 1 crosstalk regulates phagocytosis of a fungal pathogen. Nat Commun..

[61] Singh R.P., Bhardwaj A. (2023). β-glucans: A potential source for maintaining gut microbiota and the immune system. Front. Nutr..

[62] Varnosfaderani S.M.N., Ebrahimzadeh F., Oryani M.A., Khalili S., Almasi F., Heris R.M., Payandeh Z., Li C., Afjadi M.N., Bahrami A.A. (2024). Potential promising anticancer applications of β-glucans: A review. Biosci. Rep..

